# Use of nicotine replacement therapy to reduce children’s exposure to second-hand smoke in the home: a qualitative pilot study involving local community pharmacies

**DOI:** 10.1186/s12889-023-17488-5

**Published:** 2023-12-20

**Authors:** Rebecca Howell, Stephen McBurney, Giovanna Di Tano, Aileen Boags, Neneh Rowa-Dewar, Ruaraidh Dobson, Rachel O’Donnell

**Affiliations:** 1 Institute for Social Marketing and Health, Faculty of Health Sciences and Sport, University of Stirling, Stirling, FK9 4LA Scotland, UK; 2 NHS Lothian, Edinburgh, EH1 3EG Scotland, UK; 3 Usher Institute, University of Edinburgh, Edinburgh, EH8 9AG Scotland, UK

**Keywords:** Smoke-free home, Nicotine replacement therapies, Pharmacies, Second-hand smoke, Qualitative, Public health

## Abstract

**Background:**

In Scotland, and in several other countries, most second-hand smoke exposure now occurs in low-income households, where housing constraints and sole parenting often make it harder to create a smoke-free home. This pilot study provided people who smoke with a free 12-week supply of nicotine replacement therapy through local community pharmacies to reduce smoking indoors.

**Methods:**

Twenty-five parents/caregivers who smoked in the home and cared for children at least weekly were recruited via Facebook during the COVID-19 pandemic. Air quality (PM_2.5_) was monitored in participant homes for seven days before their first pharmacy visit and 12 weeks later. Qualitative interviews (*N* = 14) were conducted with 13 participants who completed the study and one who withdrew part-way through. The interviews explored views/experiences of using nicotine replacement therapy to help create a smoke-free home. Another participant took part in a shorter telephone discussion at their request, with detailed notes taken by the interviewer, because of their speech disorder.

**Results:**

Three participants reported smoking outdoors only, one of whom subsequently quit smoking. Six participants reported reduced cigarette consumption by 50% in the home, four reported no (sustained) reduction and one reported increased smoking indoors. Self-reported outcomes were not always consistent with PM_2.5_ readings. Participants’ experiences of accessing nicotine replacement therapy through community pharmacies varied. Some suggested ongoing support to use nicotine replacement products could better assist behavioural change, and that access could be streamlined by posting products to the home. Several suggested that focusing on changing home smoking behaviours using nicotine replacement therapy might facilitate a future quit attempt.

**Conclusion:**

Access to free nicotine replacement therapy for temporary use indoors may support some people who smoke to reduce children’s exposure to second-hand smoke. Our findings confirm the need to modify the intervention before undertaking a definitive trial to assess the effectiveness of this approach. This work is now underway.

## Background

The extensive range of harmful health effects associated with children’s exposure to second-hand smoke (SHS) are well established [1–3]. Creating a smoke-free home is key to reducing children’s SHS exposure, and it may also increase the likelihood of quitting smoking [4, 5] and reduce smoking uptake amongst adolescents [6, 7].

In Scotland, analysis of 2015 Scottish Health Survey data highlights a clear social inequality in children’s exposure to SHS, with 12% of children exposed to SHS at home in the most socio-economically disadvantaged areas of Scotland, compared to less than 1% of children living in Scotland’s most affluent areas [8]. This pattern has been found in several other countries including the USA, [9] Australia, [10] Germany, [11] Spain, [12] Denmark [13] and Japan [14]. The specific challenges adults who smoke living in disadvantage face in creating a smoke-free home are well documented, [15] and include sole caring for young children, and living in accommodation with limited/no access to suitable and safe outdoor space, which constrains opportunities to smoke outside [16].

There is currently no recommended approach to tackle this inequality, [17, 18] which may have been further exacerbated by the COVID-19 pandemic. In the UK, 12% of people who smoke who live with children reported smoking indoors more than they did before lockdown restrictions were imposed, [19] reinforcing the need for innovative approaches to developing interventions to better support parents/carers to create a smoke-free family home [20].

National Institute for Clinical Excellence (NICE) harm reduction guidance has identified the role of Nicotine Replacement Therapy (NRT) for temporary abstinence for people who do not want, or are not ready, to stop smoking [21]. NHS Health Scotland’s Harm Reduction Addendum [22] recommends that cessation services should advocate NRT for people who smoke, for temporary abstinence to avoid exposing others to SHS in the home. NRT could offer a means to overcome constraints such as lack of access to outdoor space, limited mobility and sole parenting. New approaches are timely and important given the Scottish Government’s ambition for a tobacco-free generation by 2034. A recent review of Scottish Government data concludes that Scotland may miss this target by 16 years in the poorest neighbourhoods [23].

Findings from a recent randomised controlled trial suggest that providing parents with a 12-week supply of NRT for temporary abstinence in the home alongside behavioural support and feedback on SHS exposure levels using air quality monitoring led to decreased SHS concentrations and cigarettes smoked in the home. Cost-effectiveness was demonstrated, but the specific effects of NRT for temporary abstinence remain unclear based on the findings from this multi-component intervention [24, 25]. To explore the sole use of NRT to create a smoke-free home, members of our research team conducted a two-phase development study [26]. Phase 1 qualitative interview findings suggested parents were open to using NRT to create a smoke-free home, viewing this as a safer option than using e-cigarettes indoors. In Phase 2, an NHS advisor discussed NRT product choice with 20 participating parents who used NRT for up to 12 weeks in the home, with ongoing support available from local community pharmacy staff. Whilst most participants reported positive changes to their smoking habits, findings relied on self-report measures of smoke-free home success, which may not be accurate if other household members smoke in the home, and may also be influenced by social desirability bias [27, 28]. In addition, the multi-step process used to access NRT was cumbersome and a barrier to engagement in some cases.

In Scotland, community pharmacies deliver a national smoking cessation service and staff are therefore trained to support patient selection of/counselling on appropriate use of NRT products. The Scottish Government has recently called for improved use of community pharmacies as the first port of call for health-related advice [29]. This study aimed to explore the feasibility of providing parents/carers with NRT for temporary abstinence in the home using a streamlined approach to NRT provision, through engagement solely with local community pharmacies. This eliminated the need for initial home visits by a trained NHS adviser (as per previous studies), as pharmacy staff discussed NRT product choice with participants.

## Methods

Study guidance for participating pharmacies was developed with the NHS Lothian Community Pharmacy Development Team. Several oral NRT products were available for use across the 12-week study (i.e. gum, spray, lozenge, tablet, inhalator) but not patches, which deliver nicotine across a 16 or 24 h period, making them unsuitable for intermittent use. Individuals could change NRT product in discussion with pharmacy staff if their first choice didn’t suit them. Once product suitability was established, participants returned to their chosen pharmacy for additional two to three week supplies of NRT as required. Pharmacy staff were advised that if a participant proactively mentioned intention to quit smoking during these visits, they should encourage them to set a quit date and support them accordingly.

### Recruitment

Parents/carers who smoked and cared for one or more children aged 16 or under at least once a week in their home were eligible to participate. More than one adult member of a household could take part provided they met these criteria. We included grandparents, aunts and uncles in our sample as they play an important role to families living in areas of high deprivation, often providing secondary care for children [30]. Exclusion criteria included individuals who were prescribed Warfarin, Clozapine, Theophylline, or Aminophylline, as NRT use in combination with any of these medications would require close blood monitoring from their GP. All individuals were recruited during the COVID-19 pandemic using paid social media (Facebook) advertising, focused on areas within a one-mile radius of the eight participating community pharmacies. All but one of the pharmacies were situated in higher deprivation postcode areas as measured by the Scottish Index of Multiple Deprivation (SIMD).

Individuals who were interested in participating submitted their contact details securely online and were telephoned by a study team member to confirm eligibility. They were emailed the participant information sheet, and after 48 h, with consent to participate provided, choice of participating pharmacy and a suitable date for baseline in-home air quality measurement were agreed.

### In-home air quality measurements

Seven-day measurement of PM_2.5_ levels in the home were made using PurpleAir PA-II-SD air quality monitors, at week one (baseline) and at week 13 (follow up, after 12 weeks of NRT use). Monitors were delivered to the home, with written instructions on self-installation in the main living area, at least 1 m from the ground and from any doors/windows. Mean PM_2.5_ concentrations were calculated for each measurement period, and baseline and follow-up mean PM_2.5_ compared. This study was not powered to find significant differences between baseline and follow-up measurements. Air quality readings were used as a means of verifying self-reported changes in home-smoking behaviours.

### Qualitative interviews

Interviews were timed to take place approximately one week after follow-up in-home air quality measurement. Interviews lasted approximately 50 min and included discussion of smoking history, NRT product use, (home) smoking patterns pre- and post-NRT use, experiences of engaging with the community pharmacy service, and the acceptability of the intervention. Each participant received feedback on their air quality levels at the end of the interview, and a £15 supermarket voucher of their choice for taking part. Fourteen interviews were conducted, including with one participant who didn’t complete the study. Interviews were audio-recorded and transcribed by a professional transcription agency. Another participant who did not complete the study took part in a short telephone discussion at their request, with detailed notes taken by the interviewer, because of their speech disorder. Ethical approval was granted by the University of Stirling’s NHS, Invasive or Clinical Research Ethics Panel (NICR 2021 0552 263). Research and Development approval was granted by NHS Lothian (project number 2021/0014).

### Qualitative analysis

The data was coded and analysed in NVivo 12 using the framework approach [31]. A thematic framework was developed (by RH and RO) to guide data management and analysis, using deductive (considering the topic guide) and inductive (reading transcripts and coding) techniques. To prepare for detailed analysis, data summaries were written in relevant cells of the framework grid (RH and RO), including hyperlinks to transcripts to facilitate data retrieval. RO reviewed all summaries to check consistency of approach and interpretation of the data. Data summaries were then used to identify high level themes before further in-depth analysis was conducted. Themes were finalised based on re-examining data and reflexive team discussions.

## Results

The characteristics of the 25 participating individuals are presented in Table 1. Fourteen grandparents, eight parents, one aunt, one uncle and one informal carer participated. Two participants lived with other adults (partners, grown-up children) who smoked but did not take part. Twenty-one participants had partial smoke-free home rules in place, with smoking permitted in specific rooms (i.e. the kitchen, living room, bedroom), and four permitted smoking anywhere in the home. During the course of the study, five participants withdrew (two changed their mind about taking part, one was hospitalised with COVID-19, two experienced a bereavement) and three became uncontactable. Two didn’t visit the pharmacy to obtain NRT because of transport and mobility issues. Fifteen participants completed the study. Participant numbers in each part of the study are illustrated in Table 2 (Tables 1 and 2 to be placed here).

**Table 1:** NRT uptake, PM_2.5_ readings and smoking-related behaviours pre-and post 12 week NRT period and self-reported outcomes

Participant ID/ Timing of Intervention	Gender/ Age	Caring status	NRT product used	Reported no. of cigarettes smoked per day				Home-smoking rule		Week 1 PM_2.5_ reading	Week 13 PM_2.5_ reading	Outcome (self report)
				Overall		In the home						
				Week 1 (baseline)	Week 13	Week 1 (baseline)	Week 13	Week 1 (Baseline)	Week 13			
**1** May-Aug ‘21	Female 59 years	Grand-parent	Lozenges	10–20	5–10	5–10	Smokes outside only	Front room. Son smokes in bedroom	Son smokes in bedroom	84	37	Smokes outside; Reduced smoking by 50% or more
**2** Jun-Sept ‘21	Female 60 + years	Grand-parent	Mouthspray, then inhalator	> 20	10	> 20	5–10	Living room, bedroom	As opposite	77	65	Reduced smoking by 50% or more
**3** May-Aug ‘21	Female 60 + years	Grand-parent	Lozenges	10–20 (roll ups)	5–8	8	2	Kitchen	As opposite	72	42	Reduced smoking by 50% or more
**4** Jun ‘21	Female (age not known)	Grand-parent	Didn’t visit pharmacy	10–20	--	10–15	--	Kitchen, Living Room	--	51	--	Withdrew
**5** Jun-Sept ‘21	Male 44 years	Parent	Mouthspray	60 (roll ups)	Quit	10	N/A	Utility room, with back door open	Smoke-free home	17	22	Smoke free home; quit smoking
**6** Jun-Sept ‘21	Male 49 years	Grand-parent	Gum, then lozenges	20	4	20	0	Living room, bedroom and study	Smoke-free home	79	29	Smoke-free home; Reduced smoking by 50% or more
**7** Jul ‘21	Female (age not known)	Grand-parent	Visited pharmacy and obtained NRT	> 20	--	> 10	--	Living room, bedroom	--	25	--	Withdrew
**8** Jul-Nov ‘21	Female 25 years	Parent	Mouthspray	20	10	10–15	4–5	Kitchen Partner smokes there too	As opposite	57	138	Reduced smoking by 50% or more
**9** Jul-Oct ‘21	Female 60 + years	Grand-parent	Inhalator, switched to lozenges	15	15	Not sure	Not sure	Kitchen, living room	Smoking more indoors - bad weather	58	134	Smoking more in the home
**10 & 11** (from one household) Aug ‘21	Female and Male (ages not known)	Aunt & Uncle	Lozenges, then inhalator	10–20	--	10–15	--	Smoking allowed anywhere in the home	--	53	125	Uncontactable for interview
			Mouthspray	5–10	--	3	--					
**12** Sept ’21 -Jan ‘22	Female 60 + years	Grand-parent	Inhalator	40	40	25	25	Living room, bedroom	As opposite	150	210	No change
**13 & 14** (from one household) Sept ‘21	Female and Male (ages not known)	Parents	Neither visited pharmacy	10–20	--	5–10	--	Kitchen and living room (when no children present)	--	36	--	Withdrew
				> 20	--	10						
**15** Sept-Dec ‘21	Male (age not known)	Parent	NRT not collected	> 20	> 20	15	15	Kitchen, living room, bedroom	As opposite	521	992	No change
**16** Sept-Dec ‘21	Female, 58 years	Grand-parent	Lozenges	> 20 (roll ups)	> 20	15	About the same	Kitchen, living room	As opposite	35	36	No change
**17 & 18** Oct ’21 – Feb ‘22	Male & Female 56 & 57 years	Grand-parents	Gum	> 20	> 20	5–6	5–6	Smoking allowed anywhere	As opposite	244	204	No change
			Didn’t visit pharmacy	10–20	10–20	8–10	8–10					Withdrew
19 Oct ’21 – Feb ‘22	Female 48 years	Parent	Inhalator	10–20	10–20			Porch with back door open	As opposite	30	24	Reduced indoor smoking
20 Nov ‘21	Female (age not known)	Grand-parent	Didn’t visit pharmacy	> 20	--	> 10	--	Living room	--	39	--	Lost to follow up
21 Nov ‘21	Female 56 years	Grand-parent	Inhalator	20	20	1–10	1–10	Spare room	As opposite	36	--	Withdrew
22 Nov ‘21	Female Age not known	Parent	Didn’t visit pharmacy	10–20	--	1–10	--	Kitchen	--	39	--	Lost to follow up
23 Jan – May ‘22	Female 53 years	Grand-parent	Spray	10–20	3	6	2	Kitchen, living room	Kitchen	70	75	Reduced smoking by 50% or more
24 Jan – Apr 22	Female 51 years	Informal Carer	Inhalator	15–20	4–5	7–10	2	Kitchen	Kitchen	185	337	Reduced smoking by 50% or more
25 Mar-Jun ‘22	Female Age not known	Parent	Didn’t visit pharmacy	20	--	10	--	Living room, bedroom	--	140	--	Lost to follow up

**Table 2:** Overall recruitment and participant engagement with the study

Participants Recruited	Air quality measured at baseline	Pharmacy visit confirmed/ NRT provided	Air quality measured at follow up	Qualitative interview completed	Participant withdrawal from the study
*N* = 25	*N* = 25	*N* = 16	*N* = 15 homes	*N* = 14 (and one short telephone discussion)	*N* = 5 withdrew (two changed their mind about taking part, one was hospitalised with COVID-19, two experienced a bereavement) *N* = 3 lost to follow up

During qualitative interviews, two participants reported creating a smoke-free home, one of whom subsequently quit smoking. A third participant reported smoking outdoors only, though their home wasn’t smoke-free as their son smoked in his bedroom. Six participants reported reducing the amount they smoked indoors, five of whom reported reduced daily smoking by 50% or more. Four participants reported no change/no sustained change to the amount they were smoking indoors. One participant noted they were smoking more often in the home. As highlighted in Table 1, self-reported outcomes were often, but not always consistent with PM_2.5_ readings. Approximately half of participants who completed the study (7/15) reported changes in home smoking that were consistent with their baseline to follow up PM_2.5_ levels. However, just under half (6/15) reported changes that were inconsistent with PM_2.5_ levels (i.e. reporting reduced smoking in the home when baseline to follow up PM_2.5_ levels had increased). A few (2/15) reported changes that were in the same overall direction as PM_2.5_ levels, though reported changes were more substantial than baseline to follow up PM_2.5_ levels suggested (i.e. reporting significant reductions in smoking in the home, whereas baseline to follow up PM_2.5_ levels reduced only slightly).

### Accessing NRT through community pharmacies

Participants had varied experiences of accessing NRT through community pharmacies. Some found staff to be very helpful:

*“They explained about each product, what each thing [NRT product] is best for, and if…if it didn’t work or anything like that, just to come back.”* (Participant 8, Parent).

Others felt that their consultations were rushed and that they were given insufficient details about different types of NRT to inform their choice of product:

*“I went and I explained [why I was there] and she says, ‘take something off the shelf.’…She wasnae helpful at all…So I just looked at two things [NRT products] and said, ‘that, and that.’…Honestly, I think she was rushed off her feet.”* (Participant 12, Grandparent).

Several participants attributed less positive experiences to community pharmacies being extremely busy during the COVID-19 pandemic (“S*moking would be the last thing on their minds at the moment.”* (Participant 23, Grandparent)). Locum staff temporarily fulfilled community pharmacy staff duties during sickness absence and this may have contributed to some occasions where staff engaging with participants were not familiar with the study:

“*The guy actually didn’t really know what I was there for when I told him, and he was a bit confused so it was a bit embarrassing and I had to explain everything to him…so I just decided to pick the [NRT] spray myself.”* (Participant 23, Grandparent).

Several spoke of the stigma associated with smoking in the home and some said they felt less comfortable about engaging in face-to-face consultations with pharmacy staff, as private consultation rooms were not in use due to COVID-19 restrictions. They suggested private discussion spaces would be important in any future study building on this work:

*“I was quite conscious of the fact I was talking about smoking in my house in public and that’s, you know, a bit embarrassing and a taboo topic…Our local pharmacy is far more intimate [than the one I visited for this study]. So you would absolutely want to do it behind a closed door there…because you know, you’re in amongst your neighbours, within ear-shot of everyone.”* (Participant 5, Parent).

Only five participants made more than one visit to their nominated pharmacy to access further NRT supplies, or to change NRT product, in most cases reporting positive experiences (“*They were really quite good with explaining things and advised on what’s the best thing to do”* (Participant 8, Parent)). However, one participant was *“too ashamed to go back”* to collect more NRT when she ran out, because she felt her initial consultation experience hadn’t gone well (Participant 9, Grandparent). Another found it difficult to visit the pharmacy because of his anxiety: “*I very seldom go out unless I really have to…Dealing with people, it’s…that’s my anxiety, which gets my stress levels up…”* (Participant 17, Grandparent).

### Use of NRT for temporary abstinence in the home

Participants reported wide ranging experiences of using NRT for temporary abstinence in the home, often reflecting on the taste or flavour of their chosen NRT product:

*“I definitely think it was working because I didn’t crave a cigarette, when I used the spray it seemed to help….But I think it was just the taste of it, I thought ‘Oh, this is vile, it’s awful!’”* (Participant 23, Grandparent).

A few participants only used their NRT product for a few days on this basis, others switched product, and a few had moved onto using e-cigarettes by the time of their interview:

*“I started off with the gum…it was too sticky for me…it was almost like tar…then I moved onto the wee tablets [lozenges]…[I] wasn’t overly keen on those, they affected my throat quite a lot…So I’ve just kind of stopped using them, didn’t use anything for about two weeks, and then I bought a vape…But by that time, I’d moved away from smoking inside anyway.”* (Participant 6, Grandparent).

Others spoke of their determination to persevere with NRT use indoors and develop new smoking habits, despite initial reservations: “*I struggled with it* [the taste] *at the beginning…but once you take it regularly, and for long periods of time you actually get used to it.”* (Participant 8, Parent). A few (including Participant 8) were still using their NRT product at the time of interview:

*“If I’m doing housework and I really want a cigarette, [before] I would have stopped and had a cigarette and a coffee. Now I go and just have a couple of puffs of that [NRT spray] and carry on what I was doing…the more the days and weeks go on, the less cigarettes I’m having…I’m hoping by the time this [NRT] packet’s finished I’ll have stopped [smoking], and if not, I’ll go and buy another packet, it’s a lot cheaper than going and buying cigarettes…and far, far healthier [laughs]*” (Participant 2, Grandparent).

### Barriers to smoking only outdoors

#### Cold/wet weather

In a few cases, participants initially reduced the amount they smoked (indoors and generally), but by the time of the interview, their daily smoking consumption had increased again to baseline levels. One individual noted that:

*“To start with, I was [smoking] in the kitchen with the back door open or in the garden, but it got to the stage where it was getting too cold, wet and miserable.”* (Participant 17, Grandparent).

Several participants suggested it was easier to smoke outside during the summer months, and this was also reflected in one account where smoking indoors had increased over the course of the study:

“*At the time when this [study] started, I was religiously going outside and having one outside, but then it got colder…and I was smoking more in the house”* (Participant 9, Grandparent).

By contrast, one participant who had successfully created a smoke-free home suggested that cold, wet weather could help him to further reduce the amount he smoked per day because he wouldn’t be motivated to smoke outside (*“I’m only having four cigarettes now. But…as it’s getting colder and wetter, I think that’s likely to just decrease.”* (Participant 6, Grandparent)).

Nuanced differences in self-reported outcomes were observed associated with timing of the intervention (see Table 1) – those who completed the study in the Spring/Summer tended to report positive changes in smoking behaviours, whereas those reporting no changes participated in the Autumn/Winter.

#### Stigma associated with Smoking in public

The stigma of smoking was another barrier for a few grandparents in particular, who were reluctant to take their smoking outside. One thought that smoking was a private activity, to be *“kept away from as many people as possible* [outdoors]*”* (Participant 9), others disliked smoking in public because their mothers had perceived this to be “*common*” (Participants 16 and 23), or because they feared people would stare at them (Participant 12).

### Benefits of this approach

Several individuals who took part had pre-existing health conditions, including asthma and chronic obstructive pulmonary disease. Participants who had changed their home smoking behaviours generally reported perceived improvements to their respiratory health:

*“So with smoking less in the house now as well, I’ve noticed that I can breathe better. And I don’t wake up in the morning coughing as much either.”* (Participant 1, Grandparent).

Some participants also reported financial benefits associated with taking part, with one using the money she would have spent on tobacco to help pay for a holiday: *“I honestly wouldn’t have been able to do it if I had been still smoking as much”* (Participant 24, Carer). Other participants found that smoking less when their children/grandchildren were present meant they spent more time together: “*It also helped me engage with the kids more*” (Participant 16, Grandparent).

Although not all participants found the NRT helpful, there was wide support for the notion of access to free NRT to enable people who smoke to reduce smoking in the home. Most interviewees reported smoking for between 30 and 55 years. Several spoke of feeling pressured by health professionals to quit smoking, suggesting that changing home smoking behaviours first might help some people who smoke to move towards quitting in the future. Reducing smoking in the home was therefore sometimes viewed as a possible stepping stone to quitting:

*“A lot of people, I think, find it hard just to give up, even if they’ve got NRT. So I think, if they* [health professionals] *motivated people and said, like, ‘well, first off, try not smoking in the house’, instead of saying, ‘just give up smoking’…then you’re cutting down slowly, and you don’t realise you’re taking longer between cigarettes…I think, yeah, people would probably be more successful in stopping in the end.”* (Participant 19, Parent).

### Possible ways to refine the intervention

Participants had several suggestions for streamlining the intervention to address barriers to uptake including reduced mobility, lone parenting and stigma associated with in-person discussions about smoking. Some suggested that NRT products could be posted to home, which could also be more convenient:

*“I think that having NRT sent to the home would probably be really good rather than having to pick it up [from the pharmacy], because…it was quite difficult at times [to pick it up] because I work long hours.”* (Participant 23, Grandparent).

Some suggested that initial NRT consultations with pharmacy staff could be conducted by telephone or online, to provide greater privacy and increase accessibility. A few grandparents suggested telephone conversations would be preferable as “*a lot of people, they’ve not got the capacity with computers, where[as] a phone call would be much easier”* (Participant 3, Grandparent). Others suggested the pandemic had increased their confidence in using online meeting systems. Participants also suggested that the provision of fortnightly (telephone or online) support to use NRT effectively could be beneficial *“to have some encouragement, you know, I think that just helps with motivation.”* (Participant 19, Parent).

## Discussion

Our findings suggest that providing individuals with access to free NRT for temporary abstinence in the home may help some to reduce or eliminate smoking in the home. This approach, which was widely accepted by study participants, builds on the findings of our first development study, with similar outcomes reported [26].

In our first development study, [26] participants often exceeded their own expectations of changing their smoking behaviours, with minimal or no support from pharmacy staff. By contrast several participants in this study felt that ongoing support for (effective) NRT use would be beneficial in future. This may to some extent reflect the composition of our sample. Several participants had smoked for between 30 and 55 years, and may therefore have found it difficult to change smoking behaviours with little to no support. Some participants only used NRT for a few days as they didn’t like the taste. This has been shown to be a potential risk factor for non-adherence to NRT in previous (cessation) studies, [32, 33] and yet most people get used to the taste of NRT after a few days [34]. Additional research is required to better understand why some participants stopped using NRT and how barriers such as taste can be better overcome. In future, issues of adherence could be addressed by provision of additional support for NRT use through community pharmacies.

In previous studies, [24, 26] access to NRT required an initial home visit from a trained NHS smoking adviser, and a subsequent visit to the local community pharmacy. We streamlined this process so that individuals discussed NRT product choice with community pharmacy staff, anticipating this would be more appealing and less burdensome based on our initial development work. In Scotland, community pharmacies remained open throughout the pandemic, offering a mobilised, accessible setting for intervention. However, the need for social distancing measures to reduce the transmission of COVID-19 often led to significant queuing outside pharmacies, and whilst staff were generally very welcoming to participants, in several cases (including when temporary staff were brought in to cover staff sickness absence) they did not have enough knowledge about the study to support participants. These experiences, alongside the preference for private consultation spaces to discuss home smoking with pharmacy staff, are similar to those described by some participants in our first development study, conducted prior to the pandemic [26, 35]. We planned to conduct interviews with pharmacy staff to learn about their experiences of taking part, but were only able to conduct one interview across the eight participating pharmacies because of competing demands on staff time. Further investigation into the challenges faced by community pharmacy staff in supporting this type of intervention would be valuable. Insights gathered could improve the intervention process, help to ensure that staff are sufficiently trained to engage effectively with participants, and identify any additional service developments required to equip community pharmacies to effectively house this intervention. Alternatively, existing NHS cessation services could be utilised, given the potential for reduced smoking in the home to lead to quitting/increased motivation to quit in some cases. NHS cessation service staff may be better placed than community pharmacy staff to support ongoing use of NRT through regular phone calls, which some participants in the current study suggested would be helpful. The inclusion of regular phone calls in future study design could also help to identify solutions to challenges to creating a smoke-free home including colder, wetter weather and perceived stigma associated with smoking in public, which are both documented in other studies [15].

Self-reported outcomes were often, but not always, consistent with PM_2.5_ readings obtained at baseline and follow-up. Whilst self-report data on smoking in the home provides useful information (see [36] for discussion regarding the reasons to collect it), it is sometimes unreliable and/or systematically biased towards under-reporting as a result of the influence of attitudes and social norms [28]. Even when individuals report home smoking rules and their associated smoking behaviours reliably, the research questions asked of them may not be sufficiently detailed to capture all scenarios which result in increased levels of exposure [36]. For example, one possible reason for discrepancies observed is smoking by other adult household members in the home [27]. Seasonal effects caused by reduced indoor ventilation during the winter months (i.e. keeping windows closed), increased burning of candles and/or an increase in wood and coal combustion for heating [37, 38] would also result in higher PM_2.5_ concentrations, which could be confused for SHS in interpretation of PM_2.5_ data [38]. If feedback on PM_2.5_ levels wrongly identifies non-SHS sources as being smoking activity this is likely to weaken the effectiveness of assessing smoke-free home interventions and result in participants questioning the validity of the measurement method [38]. It has been suggested that the combination of a biological marker of SHS (salivary cotinine, child urine cotinine, hair nicotine) and an environmental measure (air quality measurement, air nicotine monitors) used in conjunction with self-report measures enhances the reliability of assessments of SHS exposure [39]. The inclusion of a biological marker such as child salivary cotinine would overcome potential confounding environmental factors like indoor air quality changes caused by sources other than smoking and should be included in future studies.

Our findings provide additional evidence regarding the acceptability/feasibility of use of NRT for temporary abstinence in the home, which is important given there is currently no recommended approach to tackle this health inequality [17]. We successfully engaged with several participants who had smoked for between 30 and 55 years, many of whom found the idea of quitting smoking particularly difficult, but were motivated to use NRT to change their daily home smoking routines. Conducting this research during the pandemic was challenging and a larger number of participants than we anticipated withdrew or disengaged, often because of pandemic-related life events. The researcher who conducted most of the interviews was also known to participants during the study (e.g. they took informed consent and established product suitability), which may have introduced some bias.

## Conclusion

Access to free NRT for temporary use indoors may support some people who smoke to reduce children’s exposure to SHS in the home. Our findings confirm the need to modify the intervention to provide ongoing support for NRT use, easier access to NRT and assess NRT adherence more robustly, before undertaking a large definitive trial to assess the effectiveness of this approach. This work is now underway.

## Data Availability

The anonymised data underlying this article may be shared on reasonable request to the corresponding author following review.

## List of Abbreviations

SHS: Second–Hand Smoke
NRT: Nicotine Replacement Therapy
SIMD: Scottish Index of Multiple Deprivation
